# An Assessment of the Early Symptoms of Energy Deficiency as a Female Athlete Triad Risk among the Polish National Kayaking Team Using LEAF-Q

**DOI:** 10.3390/ijerph19105965

**Published:** 2022-05-13

**Authors:** Joanna Witkoś, Grzegorz Błażejewski, Marcin Gierach

**Affiliations:** 1 Faculty of Medicine and Health Science, Andrzej Frycz Modrzewski Krakow University, G. Herlinga-Grudzinskiego Street 1, 30-705 Kraków, Poland; grzegorz.blazejewski@op.pl; 2 Department of Endocrinology and Diabetology, Collegium Medicum, Nicolaus Copernicus University, Skłodowskiej-Curie Street 9, 85-094 Bydgoszcz, Poland; marcin_gierach@wp.pl

**Keywords:** female athlete triad, low energy availability in females questionnaire, kayaking, women

## Abstract

Background: Kayaking is a high intensity sport that demands high levels of aerobic and anaerobic capacity as well as a great deal of strength and endurance. The aim of this study was an assessment of the frequency of occurrence of early low energy availability symptoms using the Low Energy Availability in Females Questionnaire for women belonging to the Polish national kayaking team. Additionally, quantitative measurements of body composition and levels of calcium were performed. Methods: The study involved 33 women who were competitors in the Polish national kayaking team. Results: An analysis of the results in terms of disorders in the monthly cycle in the tested kayakers found that only five competitors, approximately 15% of the entire group, had this type of problem. Between the participants who had disorders of the menstrual cycle and those whose cycle was normal, there was only a statistically significant difference at the level of *p* < 0.001 in relation to the age of the kayakers. Conclusions: The Low Energy Availability in Females Questionnaire proved to be a useful screening tool, which allowed for the early detection of Female Athlete Triad symptoms in several young female kayakers from the Polish national team.

## 1. Introduction

The Female Athlete Triad (FAT) is the interaction of three closely related dependencies consisting of a mismatch between energy consumption and energy expenditure during physical activity. A lack of proper availability of energy may be related to an athlete’s inadequate nutrition or to eating disorders (anorexia and bulimia). Such a situation causes pathophysiology in the athlete’s body, leading to, inter alia, the absence of menstrual bleeding in women, which is the first visible symptom of reproductive dysfunction. The lack of estrogen contributes, in turn, to a decrease in bone mineral density, leading to stress fractures in women athletes [1,2,3,4,5,6].

Low energy availability (LEA) occurs when food intake is insufficient to supply the amount of energy needed to cover the requirements of both the training and maintenance of the body’s basic physiological functions. With a normal 45 kcal per kilogram of fat-free mass (FFM) per day recommended for optimal health, chronically low level energy availability, where values are below 30 kcal/kg/FFM/day, is associated with, inter alia, functional hypothalamic amenorrhea (FHA) [7].

LEA maintained over a long time leads to what is described by the International Olympic Committee (IOC) as the Relative Energy Deficiency in Sport (RED-S) syndrome. This highlights a much wider impact of energy deficiencies in the human body (athlete) than that defined for FAT, which is only a small part of the overall RED-S concept [8,9].

In the IOC consensus statement on RED-S: 2018 update [8], it was stated that ‘the syndrome of RED-S refers to impaired physiological functioning caused by relative energy deficiency and includes, but is not limited to, impairments of metabolic rate, menstrual function, bone health, immunity, protein synthesis and cardiovascular health’. The health consequences of RED-S are, therefore, much broader than just the hypothalamic-pituitary-ovary axis and bones. Moreover, it has been shown that certain psychological problems, such as mild depressive states, psychosomatic disorders, and reduced coping capacity, can be preceded or caused by RED-S [1].

FAT and RED-S can be assessed, e.g., with qualitative tools, such as a Low Energy Availability in Females Questionnaire (LEAF-Q). It is the most widely used validated screening tool to determine the risk of LEA, which it does by assessing early symptoms related to energy deficiency in women [10]. The early detection and prevention of LEA and consequent RED-S, by screening groups of athletes at particular risk of this problem, are important. Failure to detect early symptoms of LEA in an athlete leads not only to a decrease in sports performance and a worsening in the results achieved but, more importantly, to problems in the general state of health, including both physical and mental aspects [9,10,11].

Kayaking is a sport in which a person propels a boat in the water with the help of oars. In the past, it was a type of water transport, but now it is a sport discipline that appeared for the first time at the turn of the 17th and 18th centuries thanks to a regatta held in England between representatives of Oxford and Cambridge universities. The first rowing competition—Race for Doggett’s Coat and Badge—was held on the Thames in 1715. The rowing competition became so popular that it is still held today [12].

Kayaking places high demands on the strength of the muscles of the upper body and torso [13,14,15], while using a significant amount of the energy stored in the athlete’s body. Kayaking is a high intensity sport that demands a high level of aerobic and anaerobic capacity as well as a great deal of strength and endurance. Races, usually, take place over a distance of 2000 m and last from five to nine minutes; however, these distances may vary, e.g., 100 m or 500 m. Kayaking imposes no weight restrictions on competitors, unlike rowing, where there are two weight classes: openweight rowing (with no weight limits) and lightweight rowing (with a specific body weight). In order to qualify for and participate in a lightweight rowing race, competitors must meet the strict body weight conditions and weigh no more than 59 kg, with an average one-person crew weighing no more than 57 kg [13,14,15].

It should be noted that kayaking and rowing are minority sports and there is little scientific research on women who undertake this type of sporting activity. Despite the fact that the two disciplines differ slightly in terms of technique, the energy expenditure needed to propel the boat is similar in both [16]. The person sitting in the kayak is forced to overcome, by means of the movement of the oars, the aerodynamic drag acting on them and the kayak as well as the hydrodynamic drag acting on the kayak [17]. The main factors that contribute to the performance of the rower, reflected in the speed of the boat moving through the water, are the competitor’s proficiency in mastering the correct technique of cyclic rowing and the strength of their muscles [18].

The aim of this study was an assessment of the frequency of occurrence of early LEA symptoms using the LEAF questionnaire in women belonging to the Polish national kayaking team. Additionally, quantitative measurements of body composition and levels of calcium were performed. The research hypotheses were as follows. Disorders of the menstrual cycle are manifested in female kayakers. Age is a significant predictor of menstrual cycle disorders. Fat mass protects against the occurrence of menstrual cycle disorders.

## 2. Materials and Methods

### 2.1. Participants

The study involved 33 women who were competitors in the Polish national kayaking team. The study was performed in 2021, three months before the start of the kayaking season. All the competitors provided verbal consent to participate in the study. The mean age of the women studied was 20.18 years, and the standard deviation was ± 3.52 years. The average height of the kayakers was 174.76 ± 2.97 cm, body weight was 68.05 ± 4.83 kg, and body mass index (BMI) was 22.32 ± 1.47 kg/m^2^. The average number of hours a week spent on training by the participants was 10.21 ± 1.13 h. None of the women used contraceptives, either before or during the study, and their first menstruation occurred naturally between the ages of twelve and fourteen. Athletes involved in present study had the following characteristics: the majority (22) were in the Junior group, i.e., up to 19 years of age, and 3 kayakers were in the U23 group, while 8 women were in the Senior group. The kayakers had undertaken systematic training from around 12–14 years of age. They competed in categories K1, K2, and K4.

### 2.2. Body Composition Analysis

The analysis was performed using a seca device mBCA 515 medical Body Composition Analyzer. Measurements were performed in the pre-competition period, which occured three months before start of the kayaking season. The period before the start of the season was chosen due so that the competitors are not disrupted at the start of their season. The measurements were taken once in the morning on an empty stomach between 8.00 and 9.00. The time of day was chosen because it was important that the women did not drink and eat anything until after the measurements had been taken, as this could have affected the accuracy of the results obtained. The device measures the composition of the body by Bioelectrical Impedance Analysis, using a pair of electrodes for each hand and foot. The performance and accuracy of every BIA device depends directly on its validation and reference data, and seca mBCA passed an extensive scientific validation process [19,20,21,22].

### 2.3. Questionnaire

A Low Energy Availability in Females Questionnaire (LEAF-Q) was used in the study. This is a tool used for the early screening of FAT and RED-S before significant changes occur in bone mineral density (BMD) and body composition. The questionnaire examines the physiological symptoms reported by kayakers connected with LEA and includes questions sequentially on injuries, gastrointestinal, and reproductive functions. It has been validated on endurance athletes and has 90% specificity and 78% sensitivity. A result of ≥8 out of 25 questions indicates that the competitor is at risk of LEA and, as a consequence, may be at risk of developing the FAT and RED-S group of symptoms [10].

### 2.4. Statistical Analyses

Statistical analyses were performed using the SPSS 27 software program (Version 27.0, IBM Corp., Armonk, NY, USA). Test used included the following: the Mann–Whitney U (this nonparametric test was used as most variables were not normally distributed) and the chi-square test. Alpha level for all analyses was 0.05. As for effect sizes, for continuous variables, Cohen *d* was calculated as the effect size, and for categorical ones, the following was included: Cramer V. Following the guidelines for the interpretation of effect sizes, the following were assumed [23] for Cohen d: 0.2, 0.5, and 08; for Cramer V, with 1 df: 0.1, 0.3, and 0.5; and for Cramer V with 2 dfs: 0.07, 0.21, and 0.35 for small, medium, and large effect sizes, respectively.

## 3. Results

Body Composition Analysis for the entire group of women studied was as follows: the average fat mass (FM) expressed in kilograms 15.47 ± 3.23 kg, the percentage of FM at 22.44 ± 2.93%, the abdominal visceral adipose tissue (VAT) at 0.16 ± 0.09, the FFM in kilograms at 52.87 ± 2.49 kg, lean mass as a percentage at 77.57 ± 2.93%, muscle mass in kilograms at 25.48 ± 1.27 kg, total body water (TBW) at 39.36 ± 1.41%, extracellular water (EWC) at 16.29 ± 0.70%. The ratio of EWC to TBW was 41.30 ± 0.86%. The women’s calcium levels were also assessed and averaged 2.34 ± 0.05.

An analysis of the results in terms of disorders in the monthly cycle in the tested kayakers, found that only five competitors, approximately 15% of the entire group, had this type of problem. A detailed list of all the parameters obtained from the examination with the body composition analysis, with the addition of a division into women who did and those who did not have a menstrual cycle disorder, is presented in Table 1.

Between the participants who had disorders of the menstrual cycle and those whose cycle was normal, there was only a statistically significant difference at the level of *p* < 0.001 (Mann–Whitney U test) in relation to the age of the kayakers (Table 1). Women reporting disorders of the menstrual cycle were older than the women who did not report such disorders; the mean age was 26.00 ± 2.24 years vs. 19.14 ± 2.56 years, respectively. There were no other statistically significant differences, including body composition, between women who had problems with the menstrual cycle and those who did not. The effect size for age was large. For the remaining effects, their sizes were larger rather than small, but they were insignificant due to the small sample size.

An analysis of the answers provided by the kayakers to the LEAF questionnaire found that a result of ≥8 out of 25 questions was achieved by only one respondent (3.03%). This indicates that she is at risk of LEA and, as a consequence, may be at risk of developing the group of symptoms associated with FAT and RED-S. There was also one (3.03%) kayaker (3.03%) with a score of seven and three women (9.09%) with a score of six. These numbers suggest that perhaps these women may have a problem in the near future. For the remaining women, the majority of the study group, 23 out of 33 (69.70%), had had no sports injuries in the last year. Among the women who did report injuries, the number of days absence from training or participation in competition due to injuries was relatively short—for five women (50.00%), it ranged from 1 to 7 days and, for four women (40.00%), it ranged between 8 and 14 days (Table 2). Only one woman had a recovery time of 22 days or more. An analysis of the gastro intestinal function showed no significant disturbances. In the vast majority of the kayakers, there were no problems with these functions. When considering menstrual function, however, it was found that 28 women had results that were expressed as the number two. The most important questions and answers included in the LEAF questionnaire are presented in Table 2.

The vast majority of the women in 84.85% had a normal monthly cycle (Table 2). Their last period had been between 0 and 4 weeks ago, and most of the time, the periods are regular (every 28th to 34th day). However, among the kayakers who declared a lack of a menstrual cycle, the time without menstruation in four women (12.12%) was between 4–5 months, and for one woman (3.03%), it was 6 months or more. When asked about any changes to the course of their menstrual cycle could be influenced by physical activity, eight women (24.24%) said that they bleed less during menstruation (Table 2).

An analysis of the responses contained in LEAF-Q, with a direct division into women who had a problem with the monthly cycle and those who did not report such a problem, found a statistical significance at the level of *p* < 0.001 for responses regarding the time of the last menstruation, an interruption in the cycle for three or more months, and the impact of sports training on the course of the monthly cycle (Table 3). For significant effects, the size was large.

## 4. Discussion

Exercise-related issues such as irregularities in the menstrual cycle or the complete absence of monthly bleeding are significant health problems for women undertaking physical activity. The menstrual cycle disorders that occur in female athletes vary from oligomenorrhea to amenorrhea and have a direct impact not only on their sports achievements but also on the future fertility of these women [1,2,3,4,5,6].

This study did not reveal any problems with irregularities in the monthly cycle for the majority of kayakers. From the group of 33 women, only 5 (about 15%) declared that they had missed a monthly cycle for more than 3 months. Moreover, only one woman achieved a critical score of ≥8 out of 25 questions in the LEAF questionnaire, which immediately indicates that she is at risk of LEA and, as a consequence, may be at risk of developing the group of symptoms of FAT and RED-S.

The results achieved in the study are satisfactory, as they prove that there is a properly conducted training and dietary process among the Polish national kayaking team. It should be noted that the kayakers belonged to a group that was not limited by any critical body weight necessary to participate in the competition. The kayakers had an average body weight of 68.05 ± 4.83 kg, which is about 10 kg more than, for example, the required body weight of 59 kg for lightweight rowers, where other authors of studies [13] have more frequently found disorders of the menstrual cycle. It should also be noted that the energy expenditure for both types of rowing, whether it is kayaking or rowing, is very similar. Therefore, it is likely that the body weight of the kayakers in this study was the factor that helped most women maintain their monthly cycle undisturbed.

The weight category element in rowing is a risk factor for LEA and generally leads to disordered eating (DE) and a chronic low energy state in the body, which increases, inter alia, the risk of rib stress fractures in these sportswomen [24]. Stress fractures have been described as being the most widespread in endurance and aesthetic sports, i.e., those in which there is a risk of DE [25]. Studies by Walsh et al. [15] showed that lightweight rowers significantly more often reported dietary restrictions than rowers from the openweight group, 41.9% and 29.9%, respectively, and this also related to the fact that a history of DE was significantly more often reported among lightweight rowers than openweight rowers (25.7% and 13.0%). The rowers from the two groups, however, did not differ statistically significantly in terms of stress fractures and disorders of the menstrual cycle. The authors of the above study noted that out of the entire group of 78 women studied, none of them used hormonal contraception and 29.3% of the women did not have a monthly cycle. This is consistent with the studies of [25], which indicated that, in a sample of 425 female athletes, 31% of the women had disruptions to their monthly cycle. However, another study [26] found that the incidence of oligomenorrhea or amenorrhea among elite lightweight women rowers is as high as 76%. The authors of the research [22] suggest considering changes to the rules of critical body weight in lightweight rowing, as studies [13,27] have shown that rowers in this category limit their diet and use both extreme and chronic methods of weight loss.

In many women practicing sports, insufficient food intake is usually associated with a low body fat mass, which in turn leads to an inability to convert androgens to estrogens with a simultaneous decrease in the level of leptin produced mainly by adipose tissue cells. However, attention should be paid to the fact that the body of an athlete with LEA may undergo physiological adaptation to the existing energy deficiency by reducing resting energy expenditure. In such a situation, the body weight of the competitor with LEA will remain at a stable level and the measurements will be considered correct, i.e., within the norm [28].

LEA has a negative impact on many functions of a sportswoman’s body, and disruptions to the monthly cycle carry further serious health consequences mainly related to the lack of estrogen, such as bone metabolism disorders. Badania Brook et al. [29] showed that both the disruptions to the menstrual cycle and a delayed menarche are significantly associated with lower bone mineral density (BMD). It should be mentioned, however, that in the research study conducted by Maïmoun et al. [30], it was shown that high-load resistance training undertaken by female athletes has a positive effect on BMD. It was observed that women who undertook this type of training had higher bone mass compared to a control group of women of the same age and who declared the same history of menstrual cycles.

Research by [26] aimed at assessing, in conjunction with a history of monthly cycles and eating disorders, the BMD in British lightweight elite rowers and showed that women with oligomenorrhoea or amenorrhea had lower spinal Z-scores compared to those who did not have such disorders. As rib stress fractures are less common in male rowers, it is believed that low estrogen levels, associated with disruptions in the menstrual cycle, in addition to other factors related to FAT contribute to increased fractures in female rowers. Studies have found an association between the number of missed monthly cycles in female athletes and a significant decrease in BMD. Kurgan et al. [31] advised that BMD and bone mineral content remained stable throughout the season in openweight female rowers with adequate energy availability.

The authors of [29] postulate the necessity to undertake the earliest possible intervention and clinical assistance in female athletes with oligomenorrhoea or amenorrhea in order to prevent irreversible bone loss. It was also shown that the rowers who reported DE started to row at a younger age than those without DE, (10.6 ± 3.1 years vs.18.3 ± 4.9 years, respectively), which indicates that engaging in training at the highest international level at an early age is significantly associated with the risk of DE and, thus, LEA, FAT, and RED-S.

In this research study, all women experienced menarche naturally between 12–14 years of age, which, physiologically, is the correct time for this to occur [32]. The only statistically significant result recorded in this current study was that the women reporting disruptions to the menstrual cycle were older than those who reported no such disruptions, with the mean age being 26.00 ± 2.24 years vs. 19.14 ± 2.56 years, respectively. This result is somewhat surprising because the authors of [33] have shown that younger women engaging in increased physical activity are particularly vulnerable to disturbances in the menstrual cycle. For example, using a controlled exercise program with a calorie-restricted eating plan women of 25 to 40 years of age had less frequent disturbances in their menstrual cycle than women of 14–18 years in age.

Research by Badania De Souza [34], however, showed that even with regular monthly bleeding, the cycle may be disturbed because the follicular phase is prolonged, with a shortened or absent luteal phase, such a situation being known as luteal phase defects (LPDs). The consequences of this are serious, as it occurs as a result of insufficient production of progesterone (P4), giving rise to poor endometrial maturation, which is directly related to infertility or habitual spontaneous abortions. LPD is a phenomenon that occurs relatively frequently, as shown by De Souza et al. [33] in women undertaking physical activity purely at a recreational level, where the incidence of LPD was 48%, and the frequency of anovulatory cycles was as high as 78%.

Studies [35] carried out on a group of young elite female kayakers aged 16–23, showed that the mental stress experienced by sportswomen may also be a factor conducive to disturbances in the monthly cycle in sport. The research reported that the high intensity of daily training, as well as sports competition at a world level class, exposed the competitors to a high level of mental anxiety. The study showed that mental anxiety was related to menstrual irregularities. This suggests that mental stress activates the hypothalamic–pituitary–adrenal (HPA) axis, inhibits the gonadotropin-releasing hormone (GnRH) secretion, leading to an inhibition of preovulatory luteinizing hormone (LH) secretion, and inhibits ovulation as a consequence [36,37]. However, other studies found that another cause of stress among elite athletes is the need to increase body weight when it is too low. This has been associated with anger and resentment, with weight control being the most stressful aspect of sport for athletes. Stress was also caused by injuries, the lack of a fixed date for recovery and return to the sport, and the fear of a decline in sports performance and, thus, a reduction in self-esteem. The lack of monthly bleeding in sportswomen may additionally be associated with the excessive secretion of beta-endorphins [38], which causes a suppression of the HPA axis with a subsequent inhibition of LH, follicle-stimulating hormone (FSH), oestradiol (E2), and progesterone, as well as an excessive secretion of prolactin.

Eating disorders leading to LEA and subsequently to FAT and RED-S are widespread in sport and this also applies to women in the rowing sports, as shown in studies by other authors [1,2,3,4,5,6]. In order to achieve satisfactory sports results and maintain optimal health, athletes must adhere to both an established training plan and the rules of sports nutrition recommended for their given discipline. Coaches should take into account not only training strategies but also nutritional strategies for a specific sports season. The results of research suggest that deliberate weight loss by athletes combined with high-level training, which lead to disruption in the monthly cycle, may be caused by a lack of proper nutritional support provided for sportswomen by coaches and nutritionists and by a lack of adequate knowledge and understanding of the importance of proper nutrition in sport. Interestingly, about half of the rowers tested by [39] confirmed that they limit or carefully control their daily meals, which demonstrates their concerns about their own body weight and its composition. Most of the female athletes surveyed have never worked with a dietitian. The lack of nutritional knowledge certainly contributes to insufficient energy consumption in athletes. Therefore, nutritional support for female athletes, especially those at risk of FAT, would be beneficial for them. Doering et al. [34] argued that despite the fact that athletes are aware of the importance of proper nutrition in sports, they lack relevant knowledge about carbohydrate and protein intake. Heikkilä et al. [40] also found that athletes and coaches often lack nutritional knowledge. They suggested that athletes should acquire sufficient nutritional knowledge to understand the importance of food choices for competition, recovery, and overall health.

As an example, Kin et al. [41] reviewed the literature in terms of the optimal nutrition for people undertaking training for rowing, including lightweight rowers who undertake weight loss practices by limiting food and fluid consumption. A training program for rowers is high with respect to intensity, so athletes need sufficient carbohydrate intake to increase glycogen availability [42]. The average caloric intake of rowers ranges from 2600 kcal to 4900 kcal. Depending on training, it can even reach about 7000 kcal. Carbohydrates account for approximately 4.6–6.3 g/kg of the total caloric intake [43]. The recommended amount of protein in the early stage of recovery is around 20–25 g, which increases to 40 g based on factors such as age and muscle mass [44].

Knowledge about the principles of sports nutrition and the proper nutrition of an athlete enables the maintenance of optimal health, prevents energy shortages, and prepares the athlete’s body for physical exertion at the necessary level. The early detection of LEA in female athletes prevents the occurrence of FAT and further serious disorders of the body known as the RED-S syndrome. There is no standard comprehensive approach to screening and early intervention and coaches, who have daily contact with players, should be particularly aware of these issues. It is important to have a multidisciplinary approach to the problem, with both nutritional and psychological support for female athletes in order to prevent LEA and its consequences. Trainers, as recommended by the National Athletic Trainers’ Association [45], should monitor female athletes with DE and a lack of a monthly cycle, i.e., at risk of FAT, and take action for the sake of their health. Neglecting the early symptoms of LEA and FAT means that, for certain, they will develop into more serious health problems for the athlete and, consequently, herald her departure from the sport. Validated questionnaires can be used for the purpose of screening female athletes. They are easy to use and allow for early risk detection and, if necessary, the early referral of the athlete for specialist clinical observation.

Limitations and future research: The main limitation of the research was the small number of tested kayakers, but this was due to the fact that the research covered the Polish national team, i.e., women involved at the top level of a highly specialized sport. The authors of the study did not accurately assess the athlete’s performance level [46]. The research would certainly have provided a more complete picture of the disorders of the menstrual cycle if hormonal tests had been additionally performed. Despite the fact that few women had menstrual disorders, hormonal tests would have shown if the women, who had a normal menstrual cycle, had ovulatory or non-ovulatory cycles and a luteal phase defect. The authors of the study were only aware of any menstrual cycle issues relevant during the day and had no knowledge of the full history of menstrual cycle disorders in the female competitors. It would also be interesting for future directions perform hormonal tests on the kayakers as well as to analyze body compositions at different points during the preparation period for the season and even at the start of the season.

## 5. Conclusions

The Low Energy Availability in Females Questionnaire proved to be a useful screening tool, which allowed for the early detection of the Female Athlete Triad symptoms in several young female kayakers from the Polish national team. Despite the fact that the studied women, who declared disruptions to their menstrual cycle, had no issues with their body composition and weight, they had had no monthly bleeding for several months. This could lead to serious consequences, including the development of the relative energy deficiency in sport symptoms.

## Figures and Tables

**Table 1:** Differences between kayakers who have disorders of the monthly cycle (problem *n* = 5) and without such problems (no problem *n* = 28); numerical variables (Mann–Whitney U tests).

Dependent Variable	Mean		Median		Standard Deviation		*U*	*p*	Cohen d
	No Problem	Problem	NP	P	NP	P			
Age (years)	19.14	26.00	18.00	26.00	2.56	2.24	5.00	**0.001**	2.72
Height (cm)	174.68	175.20	175.00	175.00	3.19	1.30	61.00	0.648	0.17
Weight (kg)	68.60	66.70	67.55	65.35	5.36	3.05	55.50	0.467	0.37
BMI (kg/m^2^)	22.49	21.73	22.34	21.20	1.53	1.13	51.00	0.340	0.51
FM (kg)	15.70	14.04	14.98	13.39	3.34	1.61	50.00	0.315	0.52
FM (%)	22.69	21.00	21.92	20.48	3.06	1.50	43.00	0.175	0.58
VAT	0.18		0.15		0.12		-	-	
FFM (kg)	52.91	52.66	53.39	51.96	2.63	1.71	69.00	0.960	0.10
FFM (%)	77.31	79.00	78.08	79.52	3.06	1.50	43.00	0.175	0.58
MM (kg)	25.41	25.84	25.40	26.10	1.32	0.91	55.00	0.451	0.34
TBW (%)	39.40	39.62	39.60	40.10	1.44	1.22	63.50	0.744	0.16
ECW (%)	16.28	16.64	16.30	16.60	0.73	0.48	49.50	0.302	0.51
ECW/TBW (%)	41.29	42.00	41.09	42.28	1.03	0.95	40.00	0.132	0.70
Calcium (mmol/L)	2.33	2.36	2.34	2.36	0.05	0.03	49.50	0.302	0.48

**Table 2:** The most important questions of LEAF-Q and the numerical answers provided by the kayakers in the study.

Low Energy Availability in Females Questionnaire	Frequency (*N*)	Percent (%)
**1. Injuries**		
Absences from your training. or participation in competitions during the last year due to injuries		
No, not at all	23	69.70
Yes, once or twice	10	30.30
Yes, three or four times	0	0
Yes, five times or more	0	0
Numbers of days absence from training or participation in competition due to injuries		
1–7 days	5	50.00
8–14 days	4	40.00
15–21 days	0	0
22 days or more	1	10.00
**2. Gastro intestinal function**		
Do you feel gaseous or bloated in the abdomen. Also, when you do not have your period?		
Yes, several times a day	0	0
Yes, several times a week	0	0
Yes, once or twice a week or more seldom	2	6.06
Rarely or never	31	93.94
Do you get cramps or stomachache which cannot be related to your menstruation?		
Yes, several times a day	0	0
Yes, several times a week	0	0
Yes, once or twice a week or more seldom	2	6.06
Rarely or never	31	93.94
**3. Menstrual function**		
Do you have normal menstruation?		
YES	28	84.85
NO	5	15.15
When was your last period?		
0–4 weeks ago	28	100
Are your periods regular? (Every 28th to 34th day)		
Yes, most of the time	28	100
How many days do you normally bleed?		
1–2 days	3	10.71
3–4 days	25	89.29
Have you ever had problems with heavy menstrual bleeding?		
NO	28	100
How many periods have you had during the last year?		
12 or more	24	85.71
9–11	4	14.29
When did you have your last period?		
2–3 months ago	28	84.85
4–5 months ago	4	12.12
6 months ago or more	1	3.03
Have your periods ever stopped for 3 consecutive months or longer (besides pregnancy)?		
No, never	28	84.85
Yes, it has happened before	3	9.09
Yes, that’s the situation now	2	6.06
Do you experience that your menstruation changes when you increase your exercise intensity, frequency, or duration?		
YES	8	24.24
NO	25	75.76
If yes, how?		
I bleed less	8	24.24
No changes	25	75.76

**Table 3:** Responses to key components of LEAF-Q in relation to menstruation (chi-square tests).

Low Energy Availability in Females Questionnaire	Menstruation			
Absences from your training, or participation in competitions during the last year due to injuries				
*chi*^2^(1) = 0.26; *p* = 0.609, C_V_ = 0.09	No problem		Problem	
	*N*	%	*N*	%
No, not at all	20	71.43	3	60.00
Yes, once or twice	8	28.57	2	40.00
Numbers of days absence from training or participation in competition due to injuries				
*chi^2^*(2) = 0.31; *p* = 0.855, C_V_ = 0.17				
1–7 days	4	50.00	1	50.00
8–14 days	3	37.50	1	50.00
15–21 days	0	0	0	0
22 days or more	1	12.50	0	0.00
Do you feel gaseous or bloated in the abdomen; also, when you do not have your period?				
*chi^2^*(1) = 0.38; *p* = 0.538, C_V_ = 0.11				
Yes, once or twice a week or more seldom	2	7.14	0	0.00
Rarely or never	26	92.86	5	100.00
Do you get cramps or stomach ache which cannot be related to your menstruation?				
*chi^2^*(1) = 0.38; *p* = 0.538, C_V_ = 0.11				
Yes, once or twice a week or more seldom	2	7.14	0	0.00
Rarely or never	26	92.86	5	100.00
When did you have your last period?				
*chi^2^*(2) = 33.00; ***p* < 0.001**, C_V_ = 0.99				
2–3 months ago	28	100.00	0	0.00
4–5 months ago	0	0.00	4	80.00
6 months ago or more	0	0.00	1	20.00
Have your periods ever stopped for 3 consecutive months or longer (besides pregnancy)?				
*chi^2^*(2) = 33.00; ***p* < 0.001**, C_V_ = 0.99				
No, never	28	100.00	0	0.00
Yes, it has happened before	0	0.00	3	60.00
Yes, that’s the situation now	0	0.00	2	40.00
Do you experience that your menstruation changes when you increase your exercise intensity, frequency or duration?				
*chi^2^*(1) = 18.42; ***p* < 0.001**, C_V_ = 0.75				
Yes (I bleed less)	3	10.71	5	100.00
No	25	89.29	0	0.00

## Data Availability

The datasets used and/or analyzed during the current study are available from the corresponding author upon reasonable request.
